# Supplementary material for: An embryonic system to assess direct and indirect Wnt transcriptional targets
Source: Sci Rep. 2017 Sep 11;7:11092. doi: 10.1038/s41598-017-11519-z (PMC5593962; doi:10.1038/s41598-017-11519-z)
Supplement: Supplementary file 3 — Supplementary Figures [file 41598_2017_11519_MOESM3_ESM.pdf]

An embryonic system to assess direct and indirect Wnt transcriptional targets

Jahnavi Suresh[1*], Nathan Harmston[2*], Ka Keat Lim[2], Prameet Kaur[1], Helen Jingshu Jin[1], Jay B. Lusk[1], Enrico Petretto[2], Nicholas S. Tolwinski[1,3]

1 Yale-NUS College, 12 College Ave West, #01- 201, Singapore 138610

2 Duke-NUS Medical School, 8 College Road, 169857 Singapore, Republic of Singapore

3 Department of Biological Sciences[3], National University of Singapore, Block MD6, Centre for Translational Medicine, Yong Loo Lin School of Medicine, 14 Medical Drive, Level 10 South, 10-02M, Singapore 117599

Corresponding

E-mail: nicholas.tolwinski@yale-nus.edu.sg

Phone: (65) 6601 3092

* Equal Contribution

Supplemental Figures.

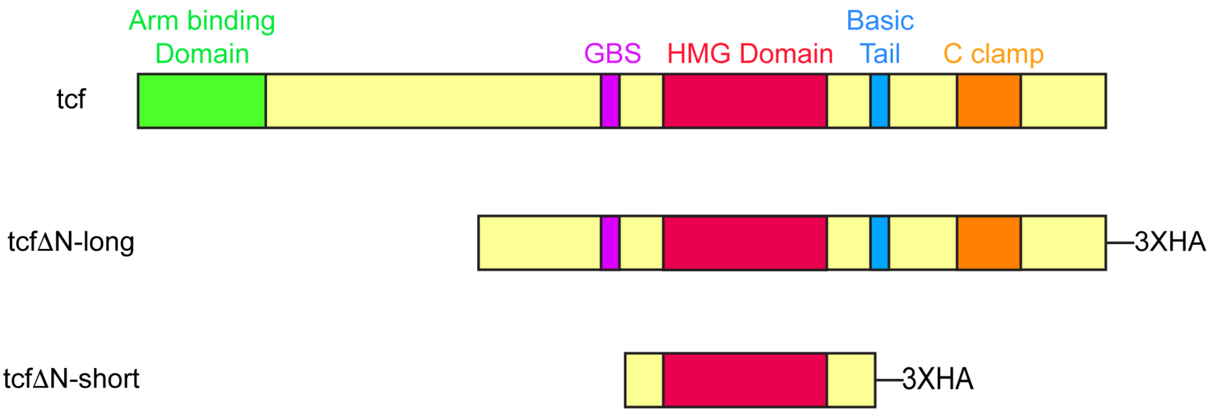

S1. **Domain structure of tcf and tcf alleles used in this study.** The schema is adapted from [1] showing the Arm or β-catenin binding domain (green), the Groucho binding sequence (GBS, purple), the high mobility group domain (HMG, red), basic tail (blue), and C clamp (orange) regions. The deleted domains as well as the HA tags are represented for the tcf constructs generated for this study.

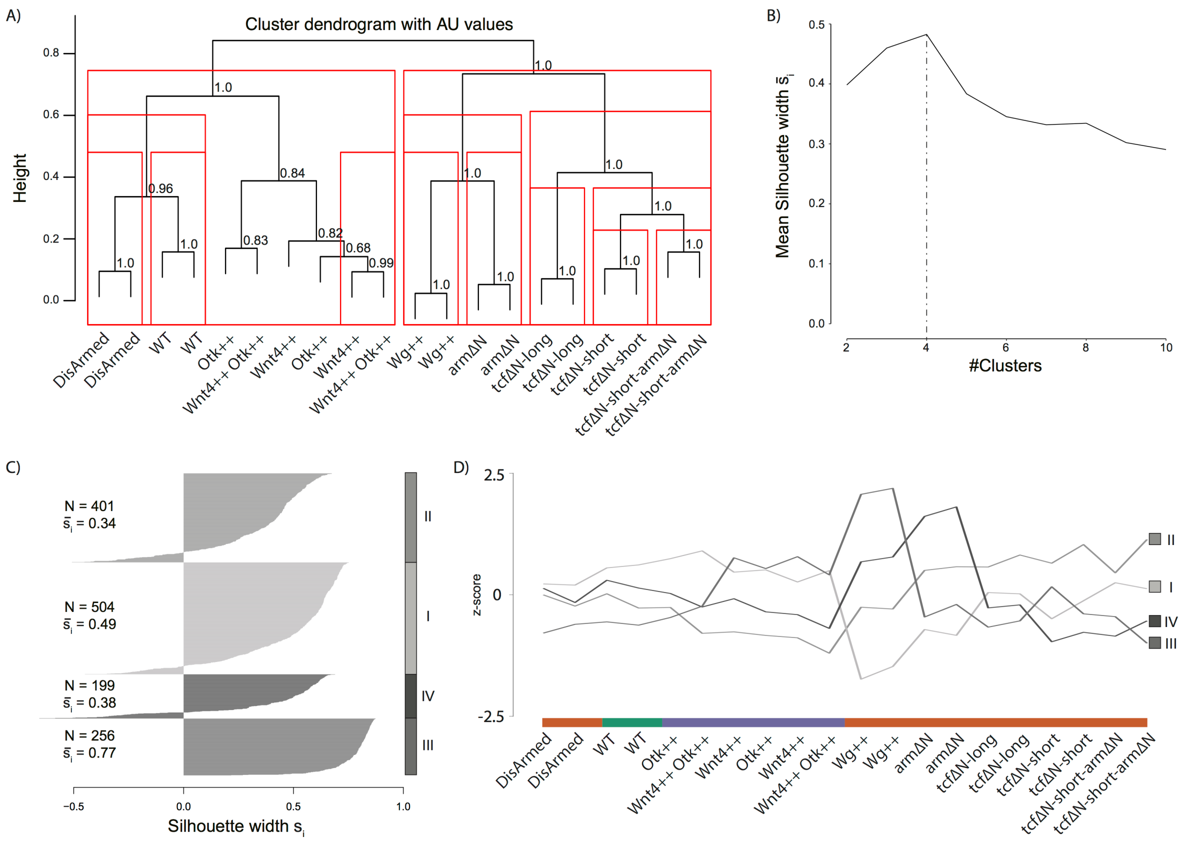

S2. **Assessment of clustering expression profiles in response to perturbing the Wnt signalling pathway.** (A) Stability of observed sample clustering (Fig. 2A) was assessed by randomly bootstrapping the data (see Methods). Support for each branch over 10000 iterations is indicated and with a red box is present to highlight that the observed clustering is not expected by chance (AU>0.95). (B) The average silhouette width was calculated across different numbers of clusters to determine the optimal choice for the number of clusters (N=4). (C) Distribution of silhouette widths across all clusters. (D) Average expression profile (z-score) for each cluster over all conditions.

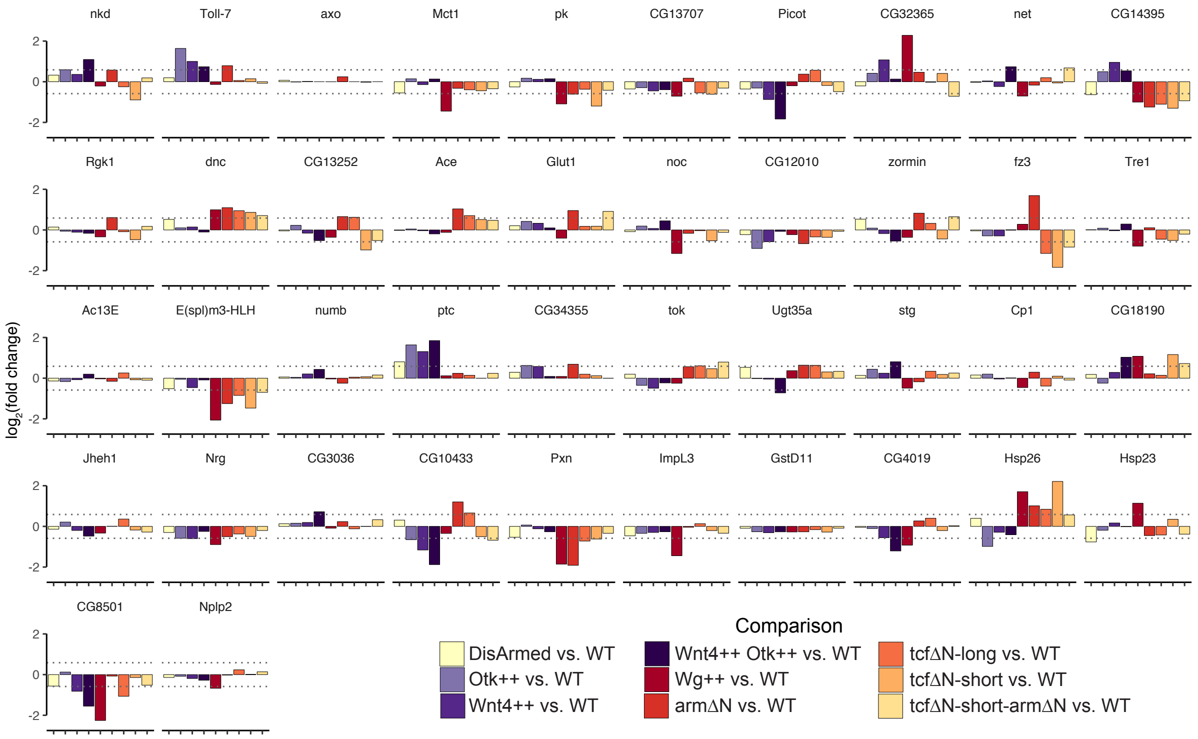

S3. **Comparison of microarray results with WNT target genes reported in Franz *et al.* (2017).** Franz *et al.* identified 51 genes in Kc167 cells as responding to growth on Wnt-conditioned media, of which 42 were identified as expressed in our microarray data. Log_2 fold changes of different perturbations compared to WT are displayed which identifies fz3, nkd and CG10433 as targets of canonical Wnt signalling and ptc as a potential target of Wnt4 [2].

Supplemental Data.

Table S1: Excel sheet with results of the differential expression analysis.

Table S2: Excel sheet containing significant enrichments for sets of differentially expressed genes

Table S3: Excel sheet with Chip qPCR primer sequences

Movie S1: Movie of *bicoid*, *nanos*, *torsolike* embryos in phase contrast

Movie S2: Movie of lightsheet microscope using Ubi-NLS-GFP and UAS-myr-Tomato[3] as nuclear and membrane markers respectively.

.

1 Cadigan, K. M. & Waterman, M. L. TCF/LEFs and Wnt signaling in the nucleus. *Cold Spring Harb Perspect Biol* **4**, doi:10.1101/cshperspect.a007906 (2012).

2 Franz, A., Shlyueva, D., Brunner, E., Stark, A. & Basler, K. Probing the canonicity of the Wnt/Wingless signaling pathway. *PLoS Genet* **13**, e1006700, doi:10.1371/journal.pgen.1006700 (2017).

3 Chen, Y. *et al.* Cell-type-specific labeling of synapses in vivo through synaptic tagging with recombination. *Neuron* **81**, 280-293, doi:10.1016/j.neuron.2013.12.021 (2014).
